# Adjuvant chemoradiotherapy vs chemotherapy for resectable biliary tract cancer: a propensity score matching analysis based on the SEER database

**DOI:** 10.1186/s40001-023-01299-w

**Published:** 2023-09-01

**Authors:** Yueting Zhu, Xia Liu, Yiyun Lin, Liansha Tang, Xianyanling Yi, Hang Xu, Yunlong Yuan, Ye Chen

**Affiliations:** 1 Department of Targeting Therapy & Immunology, Cancer Center, West China Hospital, Sichuan University, Chengdu, China; 2 Division of Abdominal Tumor Multimodality Treatment, Department of Abdominal Cancer, Cancer Center, West China Hospital, Sichuan University, Chengdu, China; 3 Graduate School of Biomedical Sciences, The University of Texas MD Anderson Cancer Center, Houston, TX USA; 4 Department of Urology, West China Hospital, Sichuan University, Chengdu, China; 5 West China Medical School, Sichuan University, Chengdu, China

**Keywords:** Biliary tract cancer, Adjuvant chemoradiotherapy, Adjuvant chemotherapy, SEER analysis

## Abstract

**Background:**

Although the role of adjuvant chemotherapy (CT) for resectable biliary tract cancer (BTC) is gradually recognized, the benefit of adjuvant chemoradiotherapy (CRT) is still controversial. Our study is designed to compare the prognosis of CRT versus CT in BCT patients.

**Methods:**

Clinicopathologic characteristics of patients with operable gallbladder cancer (GBCA), intrahepatic bile duct cancer (IHBDC), or extrahepatic bile duct cancer (EHBDC) were obtained from the Surveillance, Epidemiology and End Results (SEER) database (2004–2015). Univariate and multivariate analyses were performed to identify prognostic factors for overall survival (OS). Selection bias were reduced by propensity-score matching (PSM). Kaplan–Meier analysis was used to estimate the survival time.

**Results:**

Within 922 patients, 53.9% received adjuvant CRT, and 46.1% received adjuvant CT. Multivariate analysis showed age, primary tumor site, T stage, N stage, tumor size, number of removed lymph nodes, and treatment were independent risk factors for OS. Similar improvement of CRT on survival was identified by PSM in the matched cohort compared with CT (28.0 months vs. 25.0 months, p = 0.033), particularly in GBCA cohort (25.0 months vs. 19.0 months, p = 0.003). Subgroup analysis indicated CRT improved outcomes of patients with age ≥ 60, female, lymph nodes positive, tumor size ≥ 5 cm, and none removed lymph node diseases.

**Conclusion:**

Adjuvant CRT correlated with improved survival in patients with resected BTC compared with adjuvant CT, particularly in GBCAs. In addition, patients with age ≥ 60, female, lymph nodes positive, tumor size ≥ 5 cm, and none removed lymph node diseases may receive more benefits from adjuvant CRT.

## Highlights

- Adjuvant CRT correlated with improved survival in patients with curative resected BTC compared with adjuvant CT, particularly in GBCAs.
- Patients with age ≥ 60, female, lymph nodes positive, tumor size ≥ 5 cm, and none removed lymph node diseases may receive more benefits from adjuvant CRT.
- There were no survival differences between CRT and CT group in patients with IHBDC and EHBDC.

## Introduction

The increasing prevalence of tumors in the biliary tract (intrahepatic bile ducts, extrahepatic bile ducts, and gallbladder) is well-recognized, especially in developing countries [1]. However, its prognosis is still poor, of which the 5-year survival rates are less than 20% [2]. Biliary tract cancer (BTC) is a heterogeneous tumor with obvious differences in etiology, molecular features, treatment options, prognosis, and natural history for each subgroup [3]. Radical resection is the most effective method to cure BTC patients. Nevertheless, even with complete resection, previous studies found that about two-thirds of patients might have disease recurrence, of which 15 to 59% might have local regional recurrence [4, 5]. Therefore, in recent years, numerous efforts have been made in exploring the optimal strategy of postoperative adjuvant therapies [including radiotherapy (RT), chemotherapy (CT), and chemoradiotherapy (CRT)] for resected BTC to reduce the probability of recurrence and metastasis, so as to improve the survival. But the clinical benefit of adjuvant therapy is controversial. Until 2019, the randomized phase III BILCAP trial indicated that capecitabine adjuvant CT for 6 months following radical resection of BTC could significantly improve the relapse-free survival (RFS) and median overall survival (OS) [6], which is recommended as the new standard of adjuvant treatment in the American Society of Clinical Oncology (ASCO) guidelines [7]. Nevertheless, the lack of randomized trials and available data from small, single-institution studies led to no consensus on adjuvant CRT. Although ASCO treatment recommendations for patients with resected BTC include CT alone or in combination with RT [7], the most effective adjuvant strategy still needs to be further explored.

The specific aim of this study was to compare the impact of adjuvant CRT and CT on radically resected patients with BTC based on the Surveillance, Epidemiology and End Results (SEER) database. We evaluated the impact of CRT and CT on OS in the primary cohort. We also conducted univariate and multivariate Cox model to analyzed variables in correlation with OS. Propensity-score matching (PSM) was used to minimize the co-funding effects by nonrandom selection bias. In addition, we explored the subgroup of patients that can potentially gain benefits from adjuvant CRT. To our knowledge, it is the largest sample size analysis comparing the impact of adjuvant CRT with CT on the survival of patients with BTC based on SEER database in recent 10 years.

## Materials and methods

### Study population

**Fig. 1:**
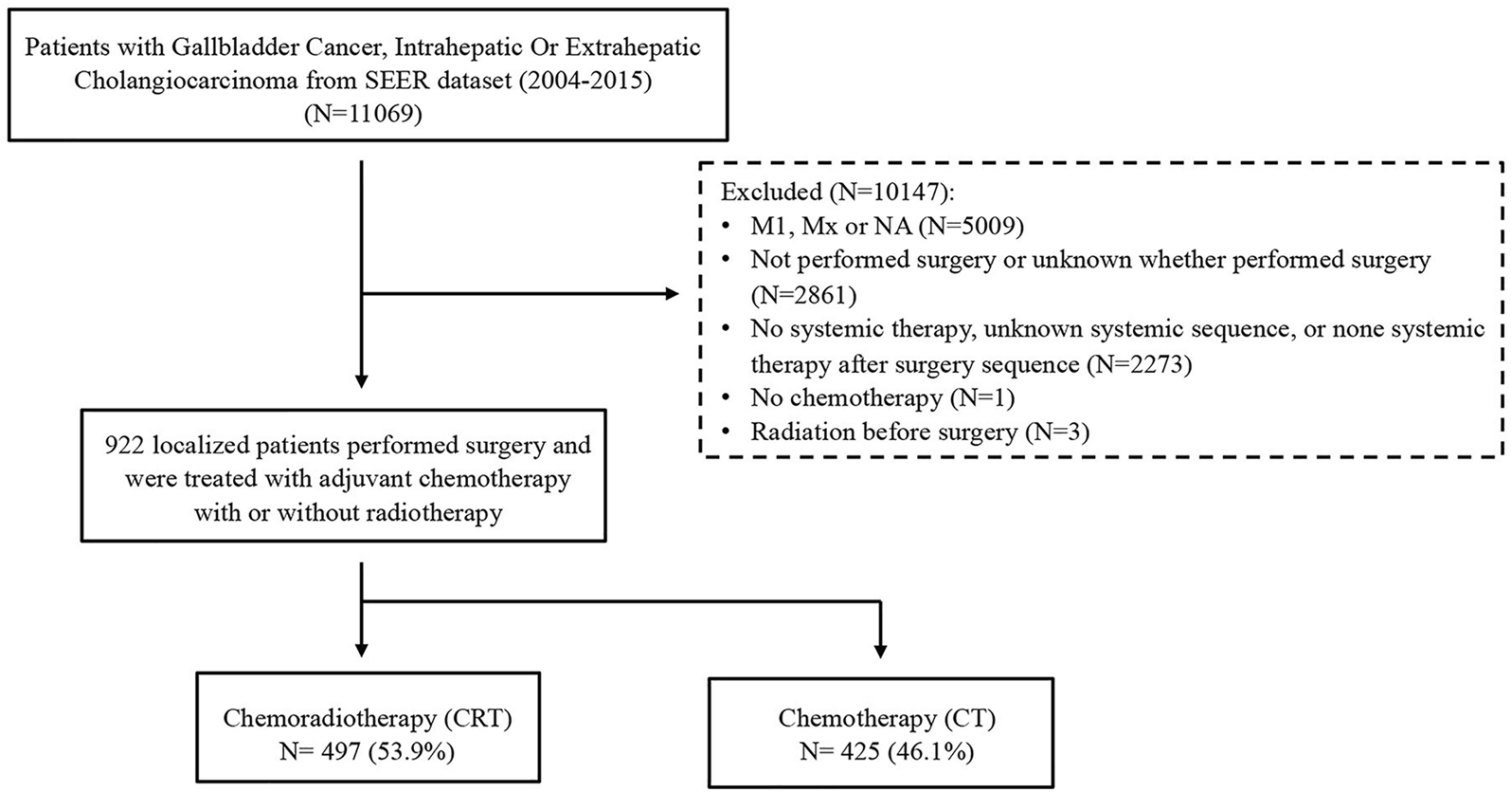
Study consort diagram

Patients with resected BTC, including intrahepatic bile duct cancer (IHBDC), extrahepatic bile duct cancer (EHBDC) and gallbladder cancer (GBCA), diagnosed between 2004 and 2015 were verified in the SEER database. Patient selection for the study cohort was depicted in Fig. 1. In brief, non-metastatic patients who performed surgical resection, followed by adjuvant CT or CRT were included. Patients who had metastatic disease, did not undergo surgery, or received neoadjuvant therapy were excluded. Clinical characteristics, including age, sex, primary tumor site, tumor size, tumor grade, T staging, N staging, M staging, number of resected lymph nodes, and type of adjuvant treatment, were obtained.

### Statistical analysis

The primary endpoint in this study was OS, which was calculated from the time of diagnosis. Continuous data were compared using the Mann–Whitney U test. Categorical variables were compared using the Chi-square test or Fisher’s exact test. OS was calculated and graphed by Kaplan–Meier methods and compared by log-rank tests. Univariable and multivariable analyses were performed using a Cox proportional hazard regression model. The variables indicating significant clinical values or significant impact (p < 0.05) were included in the multivariable Cox model. Without substitution, a 1:1 nearest neighbor PSM analysis was carried out to minimize possible confounding effects of treatment selection bias. The chi-square test was used to identify the tabulated patient characteristics after performing PSM. p < 0.05 was the threshold of significance. Analyses were performed in SPSS Statistics version 26 (IBM, Armonk, NY, USA) and figures were generated in GraphPad Prism 6 [GraphPad Software Inc., San Diego, CA, USA].

## Results

### Baseline clinical characteristics

A total of 922 patients (446 males [48.4%], and 476 females [51.6%]) were eligible and included in our study (Table 1). Adjuvant CRT was administrated in 497 patients (53.9%), and 46.1% (n = 425) received adjuvant CT. The primary tumor site was the gallbladder in 419 patients (45.4%), followed by the extrahepatic bile duct (39.5%) and intrahepatic bile duct (15.1%). According to American Joint Commission on Cancer (AJCC) staging manual, the majority of the patients were at stage T2 (35.8%) or T3 (40.1%), and had Grade II (43.9%) and III (33.8%) disease. Lots of patients did not have lymph node metastasis (N0 disease: n = 598, 53.5%), and less than 5 cm (64.9%) was the most common tumor size. Considering the number of surgically removed lymph nodes, 404 (43.8%) patients performed relatively radical surgery, with more than 4 nodes having been removed.

**Table 1:** Baseline characteristics

Characteristics	ALL (N = 922) N (%)	CRT (N = 497) N (%)	CT (N = 425) N (%)	p-value
Age				0.158^a^
< 60	286 (31.0%)	161 (32.4%)	125 (29.4%)	
≥ 60	636 (69.0%)	336 (67.6%)	300 (70.6%)	
Sex				0.096
Female	476 (51.6%)	244 (49.1%)	232 (54.6%)	
Male	446 (48.4%)	253 (50.9%)	193 (45.4%)	
Primary tumor site				** < 0.001**^**b**^
Intrahepatic bile duct	139 (15.1%)	40 (8.0%)	99 (23.3%)	
Extrahepatic bile duct	364 (39.5%)	218 (43.9%)	146 (34.4%)	
Gallbladder	419 (45.4%)	239 (48.1%)	180 (42.4%)	
T stage				0.058
T1	110 (11.9%)	47 (9.5%)	63 (14.8%)	
T2	330 (35.8%)	186 (37.4%)	144 (33.9%)	
T3	377 (40.1%)	211 (42.5%)	166 (39.1%)	
T4	105 (11.4%)	53 (10.7%)	52 (12.2%)	
N stage				**0.046**
N0	493 (53.5%)	247 (49.7%)	246 (57.9%)	
N1	422 (45.8%)	246 (49.5%)	176 (41.4%)	
Nx	7 (0.7%)	4 (0.8%)	3 (0.7%)	
Tumor size				0.059
< 5 cm	598 (64.9%)	336 (67.6%)	262 (61.6%)	
≥ 5 cm	324 (35.1%)	161 (32.4%)	163 (38.4%)	
# of removed lymph nodes				**0.046**
0	212 (23.0%)	97 (19.5%)	115 (27.1%)	
1–3	287 (31.1%)	163 (32.8%)	124 (29.2%)	
≥ 4	404 (43.8%)	228 (45.9%)	176 (41.4%)	
NA	19 (2.1%)	9 (1.8%)	10 (2.4%)	
Grade				0.101
I: Well differentiated	104 (11.3%)	60 (12.1%)	44 (10.4%)	
II: Moderately differentiated	405 (43.9%)	230 (46.3%)	175 (41.2%)	
III: Poorly differentiated	312 (33.8%)	159 (32.0%)	153 (36.0%)	
IV: Undifferentiated; anaplastic	20 (2.2%)	13 (2.6%)	7 (1.6%)	
NA	81 (8.8%)	35 (7.0%)	46 (10.8%)	

CRT: Chemoradiotherapy; CT: Chemotherapy; NA: not available

^a^t test for age. Chi-square for rest characteristics

^b^Bolded values indicate p-values < 0.05

### Survival analysis

**Fig. 2:**
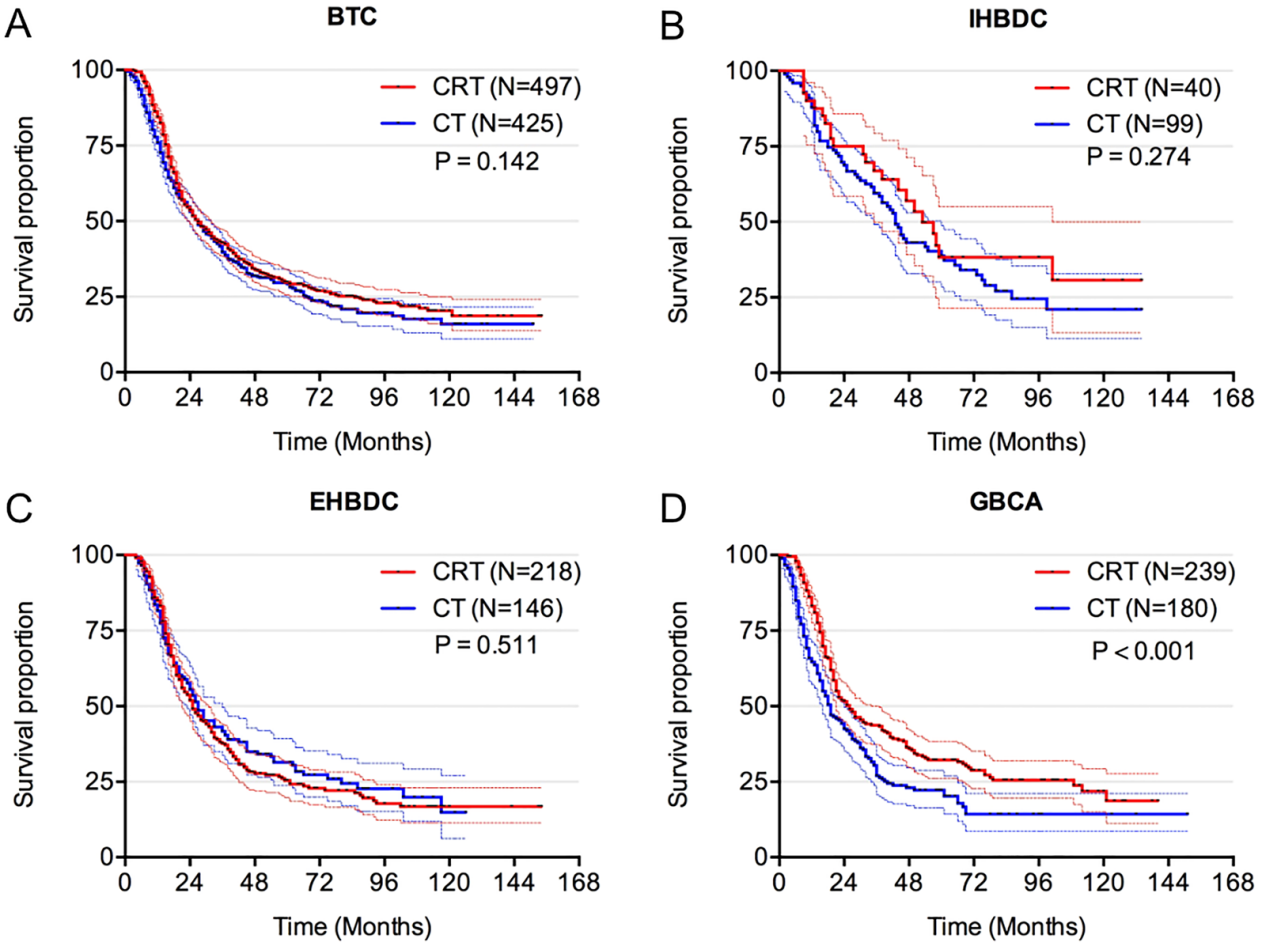
Overall survival. **a** OS for patients treated with CRT and CT in whole cohort (N = 922); **b** OS for patients treated with CRT and CT in IHBDC cohort (N = 139); **c** OS for patients treated with CRT and CT in EHBDC (N = 364); **d** OS for patients treated with CRT and CT in GBCA (N = 419). OS: overall survival; CRT: chemoradiotherapy; CT: chemotherapy; BTC: biliary tract cancers; IHBDC: intrahepatic bile duct cancer; EHBDC: extrahepatic bile duct cancer; GBCA: gallbladder cancer (GBCA)

Among the whole cohort, no favorable impact of adjuvant CRT on OS was noted compared with adjuvant CT (27.0 months vs. 27.0 months, p = 0.142) (Fig. 2a). Furthermore, in the IHBDC and EHBDC cohorts, we observed a similar trend in OS (53.0 months vs. 43.0 months, p = 0.274; 25.0 months vs. 27.0 months, p = 0.511, respectively), with no significant difference between CRT and CT strategies (Fig. 2b, c). However, in the GBCA cohort, OS for patients receiving adjuvant CRT was longer than adjuvant CT alone (26.0 months vs 19.0 months, p < 0.001) (Fig. 2d).

### Univariate and multivariate Cox analyses

In a univariable Cox model, several clinical features were associated with poor survival: age ≥ 60, primary tumor site in the extrahepatic bile duct or gallbladder, T3 or T4, N1 or Nx, tumor size ≥ 5 cm, grade II or III (Table 2). Moreover, 1–3 and ≥ 4 surgically removed lymph nodes improved outcomes with HRs of 0.688 (0.561–0.844, p < 0.001) and 0.711 (0.589–0.859, p < 0.001), respectively. Considering the clinical significance of RT and its potential association with survival, we added treatment to build our multivariable Cox model (age, primary tumor site, T stage, N stage, tumor size, number of removed lymph nodes, grade, and treatment) (Table 2). There was no statistical difference between grade and OS in multivariate analysis, while other variables were still statistically significant. Furthermore, CRT is significantly associated with a more favorable prognosis with HRs of 0.823 (0.704–0.963, p = 0.019).

**Table 2:** Univariable and multivariable cox model for OS

Parameter	Univariate		Multivariate	
	HR (95% CI)	p-value	HR (95% CI)	p-value
Age		** < 0.001**^**a**^		**0.005**
< 60	1		1	
≥ 60	1.350 (1.141,1.597)		1.282 (1.079,1.523)	
Sex		0.344		
Female	1			
Male	1.076 (0.925,1.251)			
Primary tumor site		**0.001**		**0.001**
Intrahepatic bile duct	1		1	
Extrahepatic bile duct	1.451 (1.142,1.845)	**0.002**	1.487 (1.115,1.983)	**0.007**
Gallbladder	1.557 (1.228,1.973)	** < 0.001**	1.615 (1.241,2.102)	** < 0.001**
T stage		** < 0.001**		** < 0.001**
T1	1		1	
T2	1.278 (0.972,1.681)	0.078	1.113 (0.827,1.497)	0.481
T3	1.895 (1.453,2.471)	** < 0.001**	1.619 (1.212,2.164)	**0.001**
T4	1.984 (1.444,2.725)	** < 0.001**	1.803 (1.273,2.555)	**0.001**
N stage		** < 0.001**		** < 0.001**
N0	1		1	
N1	1.400 (1.203,1.631)	** < 0.001**	1.524 (1.281,1.813)	** < 0.001**
Nx	3.172 (1.413,7.122)	**0.005**	1.863 (0.805,4.312)	0.146
Tumor size		**0.003**		**0.008**
< 5 cm	1		1	
≥ 5 cm	1.264 (1.082,1.477)		1.251 (1.060,1.477)	
# of removed lymph nodes		**0.001**		** < 0.001**
0	1		1	
1–3	0.688 (0.561,0.844)	** < 0.001**	0.585 (0.467,0.733)	** < 0.001**
≥ 4	0.711 (0.589,0.859)	** < 0.001**	0.516 (0.408,0.652)	** < 0.001**
NA	0.992 (0.594,1.655)	0.974	0.684 (0.404,1.159)	0.158
Grade		**0.006**		0.090
I: Well differentiated	1		1	
II: Moderately differentiated	1.329 (1.021,1.731)	**0.035**	1.306 (0.998,1.709)	0.051
III: Poorly differentiated	1.603 (1.224,2.099)	**0.001**	1.436 (1.091,1.889)	**0.010**
IV: Undifferentiated; anaplastic	1.636 (0.935,2.862)	0.085	1.591 (0.904,2.800)	0.107
NA	1.560 (1.105,2.202)	**0.011**	1.419 (1.000,2.015)	**0.050**
Treatment		0.142		**0.019**
CT	1		1	
CRT	0.893 (0.768,1.039)		0.823 (0.704,0.963)	

HR: Hazard ratio, CI: Confidence interval; CRT: Chemoradiotherapy; CT: Chemotherapy; NA: not available

^a^Bolded values indicate p-values < 0.05

### Survival analyses, univariate and multivariate Cox analyses in the propensity-matched cohort

**Fig. 3:**
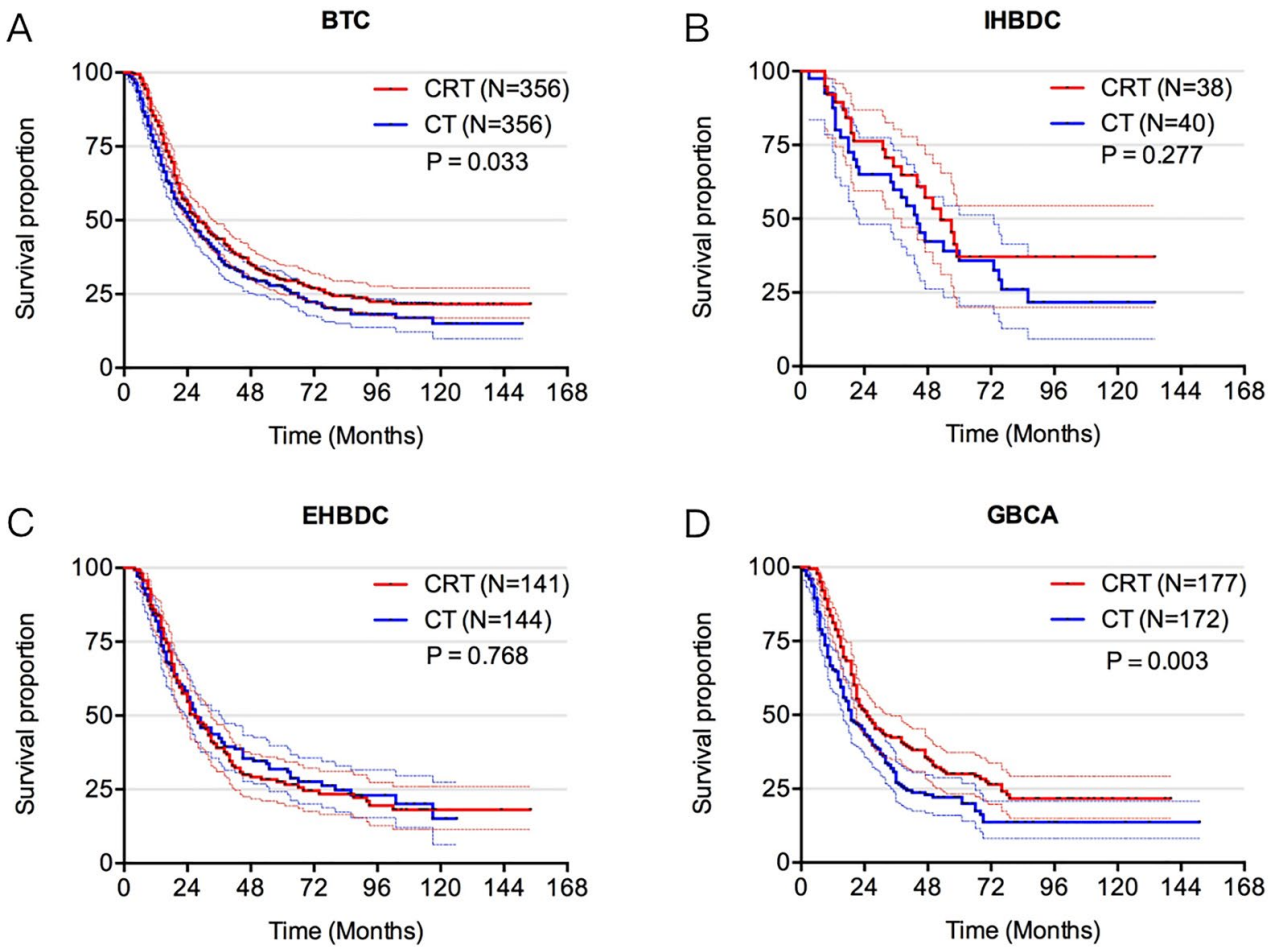
Overall survival in the PSM cohort. **a** OS for patients treated with CRT and CT in whole cohort (N = 712); **b** OS for patients treated with CRT and CT in IHBDC cohort (N = 78); **c** OS for patients treated with CRT and CT in EHBDC cohort (N = 285); **d** OS for patients treated with CRT and CT in GBCA cohort (N = 349). PSM: Propensity-Score Matching; OS: overall survival; CRT: chemoradiotherapy; CT: chemotherapy; BTC: biliary tract cancers; IHBDC: intrahepatic bile duct cancer; EHBDC: extrahepatic bile duct cancer; GBCA: gallbladder cancer (GBCA)

Regarding the imbalanced baseline characteristics between CRT and CT groups that might affect the statistical power, therefore, we used PSM to control confounding factors and build a well-balanced cohort. Patient characteristics, such as age, primary tumor site, T stage, N stage, tumor size, number of removed lymph nodes, and treatment were matched at 1:1. Table 3 shows the well-balanced baseline characteristics after matching. There was a survival benefit of CRT after PSM among the whole cohort compared with CT (28.0 months vs. 25.0 months, p = 0.033) (Fig. 3a). Among patients with IHBDC or EHBDC, a nonsignificant improvement was observed in OS with CRT versus CT (53.0 months vs. 44.0 months, p = 0.277; 27.0 months vs. 27.0 months, p = 0.768) (Fig. 3b, c). Nevertheless, in the GBCA group, those receiving CRT derived statistically greater benefits than CT alone (25.0 months vs. 19.0 months, p = 0.003) (Fig. 3d).

**Table 3:** Baseline characteristics after propensity-score matching

Characteristics	ALL (N = 712) N (%)	CRT (N = 356) N (%)	CT (N = 356) N (%)	p-value
Age				0.934
< 60	205 (28.8%)	102 (28.7%)	103 (28.9%)	
≥ 60	507 (71.2%)	254 (71.3%)	253 (71.1%)	
Sex				>0.999
Female	374 (52.5%)	187 (52.5%)	187 (52.5%)	
Male	338 (47.5%)	169 (47.5%)	169 (47.5%)	
Primary tumor site				0.926
Intrahepatic bile duct	78 (11.0%)	38 (10.7%)	40 (11.2%)	
Extrahepatic bile duct	285 (40.0%)	141 (39.6%)	144 (40.5%)	
Gallbladder	349 (49.0%)	177 (49.7%)	172 (48.3%)	
T stage				0.971
T1	72 (10.1%)	36 (10.1%)	36 (10.1%)	
T2	258 (36.2%)	132 (37.1%)	126 (35.4%)	
T3	288 (40.4%)	142 (39.9%)	146 (41.0%)	
T4	94 (13.2%)	46 (12.9%)	48 (13.5%)	
N stage				>0.999
N0	384 (53.9%)	192 (53.9%)	192 (53.9%)	
N1	322 (45.2%)	161 (45.2%)	161 (45.2%)	
Nx	6 (0.84%)	3 (0.9%)	3 (0.9%)	
Tumor size				0.755
< 5 cm	456 (64.0%)	226 (63.5%)	230 (64.6%)	
≥ 5 cm	256 (36.0%)	130 (36.5%)	126 (35.4%)	
# of removed lymph nodes				0.951
0	163 (22.9%)	84 (23.6%)	79 (22.2%)	
1–3	205 (28.8%)	100 (28.1%)	105 (29.5%)	
≥ 4	329 (46.2%)	164 (46.1%)	165 (46.3%)	
NA	15 (2.1%)	8 (2.2%)	7 (2.0%)	
Grade				0.939
I: Well differentiated	79 (11.1%)	38 (10.7%)	41 (11.5%)	
II: Moderately differentiated	306 (43.0%)	156 (43.8%)	150 (42.1%)	
III: Poorly differentiated	255 (35.8%)	127 (35.7%)	128 (36.0%)	
IV: Undifferentiated; anaplastic	14 (2.0%)	8 (2.2%)	6 (1.7%)	
NA	58 (8.1%)	27 (7.6%)	31 (8.7%)	

CRT: Chemoradiotherapy; CT: Chemotherapy; NA: not available

**Fig. 4:**
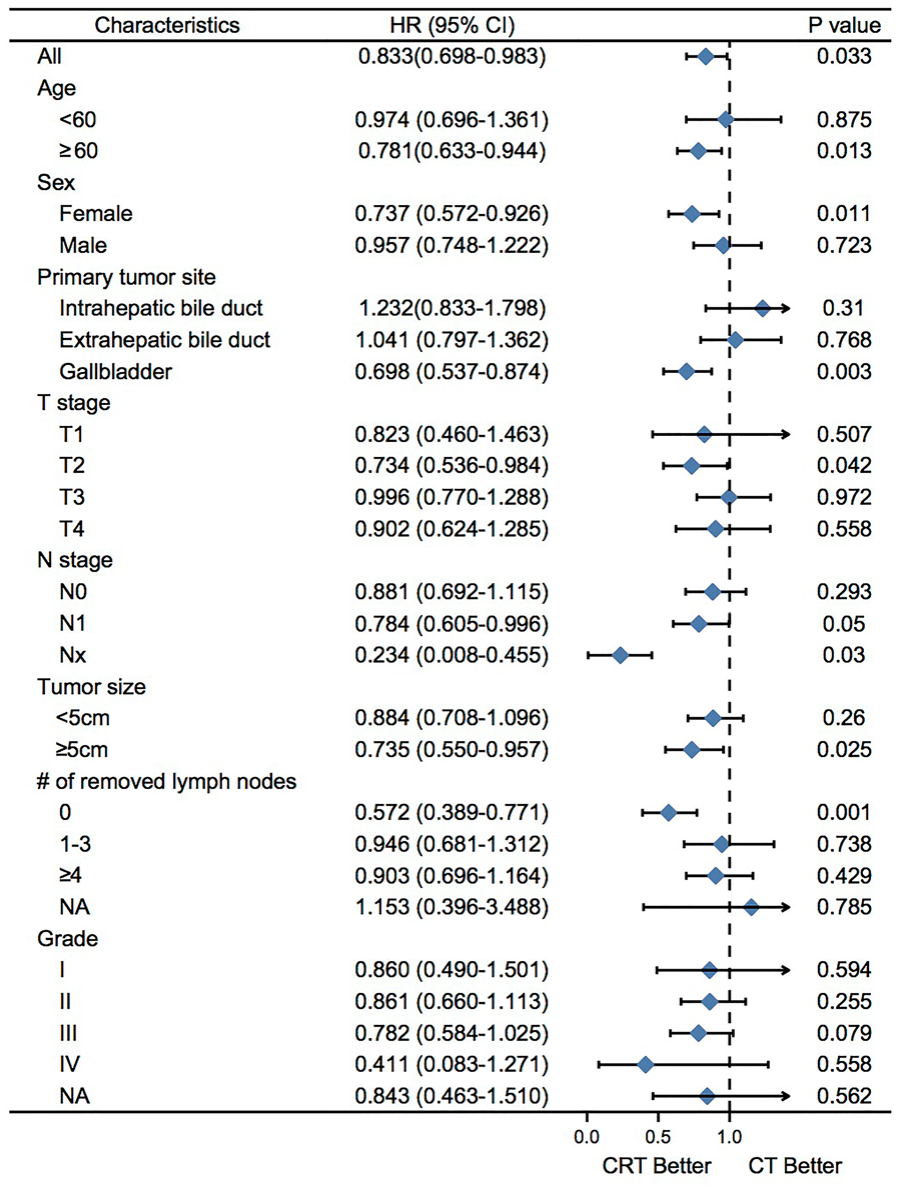
Forest plot in the PSM cohort. Forest plot for survival HRs and CIs for treatment among subgroups. PSM: Propensity-Score Matching; HR: hazard ratio; CI: confidence interval; OS: overall survival; CRT: chemoradiotherapy; CT: chemotherapy; NA: not available

The univariate and multivariate analyses of factors associated with OS in matched cohorts were summarized in Table 4. Similarly, those results revealed that age ≥ 60, primary tumor site in the extrahepatic bile duct, gallbladder, T3 or T4, N1 or Nx, tumor size ≥ 5 cm, grade II or III were related to poor prognosis, while CRT and 1–3 or ≥ 4 surgically removed lymph nodes was correlated with improved outcomes. A multivariable Cox model adjusted for these variables showed an independent adverse impact of age ≥ 60, T3 or T4, N1 or Nx, and tumor size ≥ 5 cm on OS, while CRT and 1–3 or ≥ 4 surgically removed lymph nodes indicated better survival. We explored the prognostic impact of CRT on various clinical subgroups and found that it was consistently associated with better prognosis across particular subgroups, including patients with GBCA (HR = 0.698, 95% CI: 0.537–0.874, p = 0.003), age ≥ 60 (HR = 0781, 95% CI: 0.633–0.944, p = 0.013), female (HR = 0.737, 95% CI: 0.572–0.926, p = 0.011), N1 (HR = 0.784, 95% CI: 0.605–0.996, p = 0.050), Nx (HR = 0.234, 95% CI: 0.008–0.455, p = 0.030), tumor size ≥ 5 cm (HR = 0.735, 95% CI: 0.550–0.957, p = 0.025), and none removed lymph nodes disease (HR = 0.572, 95% CI: 0.389–0.771, p = 0.001) (Fig. 4).

**Table 4:** Univariable and multivariable cox model for OS in the propensity-score matching cohort

Parameter	Univariate		Multivariate	
	HR (95% CI)	P-value	HR (95% CI)	P-value
Age		**0.006**^**a**^		**0.025**
< 60	1		1	
≥ 60	1.322 (1.084,1.613)		1.264 (1.030,1.550)	
Sex		0.305		
Female	1			
Male	1.095 (0.921,1.303)			
Primary tumor site		**0.002**		**0.002**
Intrahepatic bile duct	1			
Extrahepatic bile duct	1.531 (1.105,2.120)	**0.010**	1.534 (1.065,2.209)	**0.021**
Gallbladder	1.708 (1.241,2.351)	**0.001**	1.794 (1.282,2.510)	**0.001**
T stage		** < 0.001**		** < 0.001**
T1	1		1	
T2	1.240 (0.884,1.741)	0.213	1.006 (0.700,1.445)	0.975
T3	1.824 (1.313,2.536)	** < 0.001**	1.485 (1.047,2.106)	**0.027**
T4	1.964 (1.344,2.869)	** < 0.001**	1.840 (1.221,2.773)	**0.004**
N stage		** < 0.001**		** < 0.001**
N0	1		1	
N1	1.403 (1.178,1.672)	** < 0.001**	1.507(1.237,1.837)	** < 0.001**
Nx	3.051 (1.355,6.870)	**0.007**	1.958 (0.837,4.579)	0.121
Tumor size		**0.017**		**0.038**
< 5 cm	1		1	
≥ 5 cm	1.242 (1.040,1.485)		1.222 (1.011,1.477)	
# of removed lymph nodes		**0.004**		** < 0.001**
0	1		1	
1–3	0.745 (0.589,0.943)	**0.014**	0.655 (0.506,0.848)	**0.001**
≥ 4	0.668 (0.536,0.831)	** < 0.001**	0.507 (0.387,0.663)	** < 0.001**
NA	0.950 (0.556,1.621)	0.850	0.698 (0.402,1.212)	0.201
Grade		**0.004**		0.153
I: Well differentiated	1		1	
II: Moderately differentiated	1.375 (1.017,1.860)	**0.039**	1.307 (0.959,1.782)	0.090
III: Poorly differentiated	1.680 (1.239,2.278)	**0.001**	1.452 (1.062,1.986)	**0.020**
IV: Undifferentiated; anaplastic	2.386 (1.212,4.697)	**0.012**	1.854 (0.932,3.688)	0.078
NA	1.608 (1.083,2.389)	**0.019**	1.371 (0.916,2.050)	0.125
Treatment		0.082		**0.032**
CT	1		1	
CRT	0.857 (0.721,1.020)		0.824 (0.691,0.984)	

HR: Hazard ratio, CI: Confidence interval; CRT: Chemoradiotherapy; CT: Chemotherapy; NA: not available

^a^Bolded values indicate p-values < 0.05

## Discussion

In past years, gemcitabine based postoperative adjuvant CT regimen did not obtain positive results in two phase 3 studies of PRODIGE-12/ACCORD-18 [8] and BCAT [9]. Until recently, in the phase III BILCAP trial, prespecified per-protocol analysis showed patients received capecitabine as adjuvant therapy had better OS and RFS compared with those with observation (53 months vs. 36 months, p = 0.028; 24.4 months vs. 17.5 months, p = 0.033) [6]. According to the results of BILCAP trial, adjuvant CT with capecitabine has become the standard of care for patients with BTC after radical surgical resection [6]. However, based on the adjuvant CT, controversy continues over the role of additional RT in BTC due to lacking large, prospective clinical trials. In our retrospective analysis, compared to adjuvant CT, CRT was expected to improve survival significantly for BTC, particularly GBCA, whereas no significant benefit in patients with IHBDC or EHBDC has been observed. Moreover, specific sub-cohorts of patients, age ≥ 60, female, lymph nodes positive, tumor size ≥ 5 cm, and none removed lymph nodes disease could benefit from adjuvant CRT after PSM.

As early as in 2012, a pivotal meta-analysis evaluated CT, RT, or CRT compared with surgery alone for 6712 BTC patients from twenty studies, indicating that receiving adjuvant CT or CRT in resected BTC showed a greater survival benefit than RT alone (OR, 0.39, 0.61, and 0.98, respectively; p < 0.02), especially in those with LN-positive and R1 disease [10]. In 2015, SWOG S0809, a prospective single-arm phase II study, tested the adjuvant CT and CRT in patients with resected EHBDC or GBCA and demonstrated promising therapeutic efficacy [11]. In this analysis, the median OS was 35 months, with 2-year survival of 65%. For patients with R0, the median survival time was 34 months (2-year survival rate, 67%), whereas the median OS was 35 months for R1 patients with 2-year survival rate of 60% [11]. However, the relative value of postoperative adjuvant CRT versus CT is not clear. Several small retrospective studies have illustrated that CRT could enhance outcomes in patients with BTC compared with CT. Patients with EHBDC, hilar cholangiocarcinoma or nonhilar extrahepatic bile duct cancer (NH-EHBDC) also experienced survival benefits from CRT, particularly those with a high risk of tumor relapse [12, 13]. Moreover, Kim et al. [14] analyzed 92 patients who had undergone curative resection for BTC and received adjuvant CRT or CT. In this series, adjuvant CRT had numerically higher OS (30.1 months vs. 26.0 months, p = 0.222) and significantly better RFS (13.8 and 11.2 months, p = 0.014) than CT. Baeza et al. [15] analyzed 49 macroscopically complete resected GBCA patients treated with adjuvant CRT, and reported a favorable 5-year OS of 52%. These findings highlight the critical role of adjuvant CRT in patients who performed surgical resection for BTC. However, several studies have indicated that there were no significant differences in OS between adjuvant CT and CRT treatment [16, 17]. One study reported that the CRT group had comparable DFS (p = 0.089) and OS (p = 0.299) compared to the CT group in perihilar cholangiocarcinoma (PHC) patients with R1 resection [16]. Wan et al. [17] suggested adjuvant CT and CRT have similar effects on stage II GCB patients, and neither improves survival. Given the inconsistent results and small sample size of these data, we conducted this present study to compare adjuvant CT and CRT in patients with resected BTC based on SEER database.

Liver was the most common relapse site for BTC, followed by a local site, peritoneum and abdominal lymph nodes [9]. Both GCBA and EHBDC show high incidences of local invasion, lymph node metastasis, and distant metastasis [18, 19], while IHBDC tends to be predominantly intrahepatic recurrence, which possibly result in limited benefit from the additional adjuvant RT. Those may explain why CRT obviously improved outcomes of GCBA patients but had no significant benefit in patients with IHBDC or EHBDC in our report. In keeping with our findings, a recent nomogram model built from SEER GBCA database indicated that CRT outperformed CT for all patient subsets, those patients with T2 or node-positive disease were predicted to have survival advantage from CRT [20]. Retrospective studies also showed that resected GBCA patients with lymph nodes positive or R1 resections might derive the greatest benefit from adjuvant CRT [13, 21]. Chang, W.I. et al. [13] demonstrated that in patients with NH-EHBDC, those who had high-risk features such as nodal involvement, pT3 stage, poorly differentiated tumor, tumor size ≥ 5 cm, or R1 resection experienced a survival benefit from adjuvant CRT. Therefore, adjuvant CRT should be recommended for specific subsets of patients following surgical resection.

Nonetheless, our study still has a few limitations. Firstly, as a retrospective study of the database, treatment and selection bias cannot be avoided. Therefore, PSM analysis was used to reduce the bias. Secondly, SEER lacks information on disease recurrence; thus, we chose OS as our primary endpoint. Furthermore, the SEER database cannot provide detailed information about CT, including specific CT regimens and cycles and RT, including the RT dose, target area of RT and RT technique. In addition, SEER does not have information regarding performance status, therapeutic toxicity, and complications. We believe this diversity may represent more realistic survival situation in real world. Despite those limitations, SEER provides us with an extensive series of BTC patients, making it possible to make clinical decisions for rare tumors based on available large cohorts.

In conclusion, our analysis demonstrates that patients with BTC, particularly those with GBCA, age ≥ 60, female, lymph nodes positive, tumor size ≥ 5 cm, none removed lymph nodes disease may derive the most significant benefit from adjuvant CRT. There were no survival differences between CRT and CT group in patients with IHBDC and EHBDC. More large-scale prospective randomized clinical trials are warranted to investigate the effect of adjuvant CRT on BTC.

## Data Availability

Not applicable.
